# Study protocol: systematic review and meta-analysis of randomized controlled trials in first-line treatment of squamous non-small cell lung cancer

**DOI:** 10.1186/2046-4053-3-102

**Published:** 2014-09-16

**Authors:** Amy M DeLozier, Jacqueline Brown, Fanni Natanegara, Luping Zhao, Zhanglin Lin Cui, Stephen L Able, Lee Bowman, Joseph Treat, Lisa M Hess

**Affiliations:** 1 Eli Lilly and Company, Indianapolis, IN, USA; 2 Eli Lilly and Company, Erl Wood Manor, Windlesham, Surrey, UK

**Keywords:** Non-small cell lung cancer, Chemotherapy, Non-squamous, Cancer, Meta-analysis, Network meta-analysis

## Abstract

**Background:**

There is a high unmet need for effective treatments for patients with squamous non-small cell lung cancer (NSCLC). Eli Lilly and Company is conducting a phase III, randomized, multicenter, open-label study of gemcitabine plus cisplatin plus necitumumab (GC + N) versus gemcitabine plus cisplatin (GC) for the first-line treatment of patients with stage IV squamous NSCLC. Given GC is not the only treatment commonly used for the treatment of squamous NSCLC, this study was designed to compare the survival, toxicity, and quality of life outcomes of current treatment strategies for squamous NSCLC in the first-line setting.

**Methods/Design:**

A systematic review and meta-analysis (including indirect comparisons) of treatments used in squamous NSCLC will be conducted to assess the clinical efficacy (overall and progression-free survival), health-related quality of life (HRQoL), and safety (grade 3–4 toxicity) of GC + N compared to other treatments used in squamous NSCLC. PRISMA (Preferred Reporting Items for Systematic Reviews and Meta-Analyses) guidelines will be followed for all aspects of this study. A systematic literature review will be conducted to identify randomized controlled trials evaluating chemotherapy treatment in first-line NSCLC. Eligible articles will be restricted to randomized controlled trials (RCTs) among chemotherapy-naïve advanced NSCLC cancer patients that report outcome data (survival, toxicity, or quality of life) for patients with squamous histology. Following data extraction and validation, data consistency and study heterogeneity will be assessed. A network meta-analysis will be conducted based on the available hazard ratios for overall and progression-free survival, odds ratios for published toxicity data, and mean difference of HRQoL scales. Sensitivity analyses will be conducted.

**Discussion:**

This is a presentation of the study protocol only. Results and conclusions are pending completion of this study.

**Systematic review registration:**

PROSPERO CRD42014008968

## Background

Lung cancer is the leading cause of cancer-related deaths worldwide, accounting for 1.3 million deaths annually [1]. It is defined as cancer that forms in the tissues of the lung, usually in the cells lining air passages, and is divided into two main subtypes: small cell lung cancer (SCLC) and non-small cell lung cancer (NSCLC). NSCLC is the predominant subtype form and accounts for about 85% of all lung cancers [2]; it is further divided by cell histology into adenocarcinoma, squamous cell carcinoma, and large-cell carcinoma, with adenocarcinoma the currently predominant histology. Although the overall age-adjusted incidence rates for lung cancer are declining in many developed nations, lung cancer remains the leading cause of cancer-related deaths worldwide with an overall 5-year survival rate of about 15% [3], resulting in a significant disease burden worldwide.

The treatment of lung cancer is based on the type and stage of tumor, as well as the patient’s general medical condition. For patients diagnosed with early stage disease (i.e., stages I and II), surgery offers the best option for survival and cure. Adjuvant chemotherapy is increasingly used in those with stage II disease and occasionally for those with stage IB, depending on the size of the tumor. For those with stage III lung cancer, chemoradiotherapy alone or in addition to surgery is used to treat patients; however, while treatment is administered with a curative intent, the 5-year survival for patients with regional disease is approximately 26%, which decreases to 3.9% for patients with metastatic disease [3]. Treatment for patients with advanced disease tends to be palliative, although extension in survival may be achieved. The standard first-line drug treatments for advanced NSCLC, neoadjuvant, adjuvant, or chemoradiotherapy, are generally based on the combination of a second- or third-generation cytotoxic drug with a platinum agent (cisplatin or carboplatin).

There are many drug therapies available for treatment of NSCLC; however, not all current therapies are suitable for use in tumors of all histologies. The results of clinical trials have indicated that drugs such as pemetrexed have greater efficacy among patients with adenocarcinoma than those with other NSCLC histologies (e.g., squamous cell carcinoma) [4]. Other newer agents, such as bevacizumab, are indicated for adenocarcinoma because of higher toxicities observed in patients with squamous histology [5]. Drugs such as erlotinib and gefitinib are not restricted by histology, but have greater efficacy among patients with epidermal growth factor receptor (EGFR) mutations [6,7]. The frequency of EGFR mutations in patients with squamous cell carcinoma, as opposed to those with adenocarcinoma, is very low [8]. Therefore, histology-specific treatment options are limited for patients with squamous cell carcinoma, which accounts for about 25% of all non-small cell lung cancers [9].

There is thus a high unmet need for effective treatments for patients with squamous NSCLC, as disease burden is large and there is currently a lack of targeted drug therapies for NSCLC squamous cell tumors. Eli Lilly and Company is currently developing necitumumab as a first-line treatment in patients with stage IV squamous NSCLC. The current phase III study (ClinicalTrials.gov identifier: NCT00981058) is a randomized, multicenter, open-label study of gemcitabine-cisplatin chemotherapy plus necitumumab (GC + N) versus gemcitabine-cisplatin (GC) chemotherapy alone in first-line treatment of patients with stage IV squamous NSCLC. The target patient population for this trial is comprised of male and female patients with histologically or cytologically confirmed, advanced squamous NSCLC, previously untreated for metastatic disease.

The purpose of this systematic literature review and meta-analysis is to compare survival, toxicity, and quality of life outcomes of current treatment strategies with necitumumab among patients with squamous NSCLC.

## Methods/Design

This systematic literature review and meta-analysis (including indirect comparisons) will be conducted of treatments used in squamous NSCLC to assess the clinical efficacy, quality of life, and safety of GC + N compared to other treatments used in squamous NSCLC. To complete this objective, the following specific aims will be pursued:

1. To conduct a systematic literature review of randomized trials of all relevant treatments used for the first-line treatment of advanced squamous NSCLC;

2. To extract relevant data from the relevant published literature;

3. To perform indirect and direct comparisons of GC + N to all identified comparators for the following outcomes:

3.1 Overall survival;

3.2 Progression-free survival;

3.3 Toxicity; and

3.4 Quality of life

### Search strategy

Searches will be conducted in PubMed, Ovid/MEDLINE, and Embase using free text and controlled vocabulary terms (MeSH). Studies published prior to 1995 will be excluded as NSCLC histology was not clearly differentiated at that time. Studies not published in English will be excluded. Comparisons will be made across all regimens and not just limited to "add-on" therapies. Tables 1, 2, and 3 detail the specific search strategies for PubMed, Ovid, and Embase, respectively.

**Table 1:** PubMed search strategy

**PubMed search**			
**Category**	**Search**	**Query**	**Items found**
Disease terms	#1	"carcinoma, non small cell lung/drug therapy" [MeSH Terms]	11,135
Design terms	#2	Randomized Controlled Trials as Topic [MeSH Major Topic]	12,361
	#3	"randomized controlled trials as topic" [MeSH Terms]	85,095
	#4	Random Allocation [MeSH Terms]	76,843
	#5	double blind method [MeSH Terms]	118,616
	#6	"controlled clinical trial" [Publication Type]	85,642
	#7	"randomized controlled trial" [Publication Type]	344,749
	#8	"clinical trials as topic" [MeSH Terms]	263,513
	#9	"clinical trial" [Publication Type]	709,361
	#10	((#2 or #3 or (#4 and (#5 or #8 or #9)) or #6 or #7))	509,729
	#11	((randomization and control and clinical and trial))	10,468
	#12	(((randomised and control and clinical and trial) or (randomized and control and clinical and trial)))	108,134
	#13	((((double or single or triple or treble) and (blind* or mask*) and (random*))))	136,554
	#14	(((random and allocat*) and control* and trial))	4,775
	#15	(#12 or #13 or #14)	214,899
	#16	(#10 or #15)	527,440
	#17	(#1 AND #16)	2,065
Exclusion terms	#18	Case report [Title/Abstract]	195,029
	#19	Review [Publication Type]	1,765,829
	#20	Letter [Publication Type]	794,012
	#21	"systematic review" [Title/Abstract]	39,341
	#22	"clinical review" [Title/Abstract]	3,144
	#23	(#18 OR #19 OR #20 OR #21 OR #22)	2,734,788
	#24	(#17 NOT #23)	1,579
Year and language terms	#25	((#24) AND ("1995" [Date - Publication]: "2013" [Date - Publication])) AND English [Language]	1,217

**Table 2:** Embase search strategy

**Embase search**			
**Category**	**Search**	**Query**	**Hits**
Design terms	#1	"randomized controlled trial (topic)"/exp	37,931
	#2	"randomized controlled trial"/exp	337,523
	#3	"randomization"/exp	61,698
	#4	"double blind procedure"/exp	115,032
	#5	[controlled clinical trial]/lim	511,199
	#6	[randomized controlled trial]/lim	337,523
	#7	"clinical trial"/exp	961,450
	#8	"clinical trial (topic)"/exp	73,222
	#9	#1 OR #2 OR #3 OR #4 OR #5 OR #6	1,088,520
	#10	singl*: ab,ti OR doubl*: ab,ti OR treb*: ab,ti OR tripl*: ab,ti AND (blind*: ab,ti OR mask*: ab,ti)	174,394
	#11	"placebo"/exp	236,244
	#12	random* AND (clinical OR control*) AND trial OR (placebo* AND ("randomly allocated" OR (allocated AND random*)))	504,649
	#13	(#7 OR #8) AND (#10 OR #11 OR #12)	513,722
	#14	#9 OR #13	705,098
Exclusion terms	#15	[conference review]/lim	3,863
	#16	"case report":ab,ti	256,868
	#17	[review]/lim	2,026,389
	#18	[letter]/lim	827,115
	#19	"phase 1 clinical trial"/exp	24,619
	#20	[short survey]/lim OR "historical article": ab,ti	514,714
	#21	"systematic review": ab,ti	78,920
	#22	"clinical review": ab,ti	3,904
	#23	#15 OR #16 OR #17 OR #18 OR #19 OR #20 OR #21 OR #22	3,628,070
	#24	#14 NOT #23	424,301
Rx terms	#25	"drug therapy"/exp/mj	574,153
	#26	"treatment response"/exp/mj	2,763
	#27	"treatment outcome"/exp/mj	28,936
	#28	"drug efficacy"/exp/mj	153,753
	#29	"outcome assessment"/exp/mj	9,603
	#30	chemothera* OR ("drug"/exp/mj AND thera*) OR antineoplastic* OR palliat* OR standar* NEAR/2 care OR support* NEAR/2 care OR "best supportive care" OR best?support* NEXT/2 care	1,895,030
	#31	"radiotherapy"/exp	367,367
	#32	#25 OR #26 OR #27 OR #28 OR #29 OR #30 OR #31	2,609,134
	#33	#23 AND #31	88,937
Disease terms	#34	((lung$ OR pulmon$) NEAR/5 (adenocarcinom$ OR squamous OR "large cell" OR "non-small cell")): ab,ti	44,817
	#35	"lung non small cell cancer"/exp	52,027
	#36	metastatic: ab,ti	181,820
	#37	advanced: ab,ti	320,393
	#38	stage 3: ab,ti	61
	#39	"Stage 3": ab,ti	8,708
	#40	stage 4: ab,ti	32
	#41	"stage 4": ab,ti	5,270
	#42	stage iii: ab,ti	30
	#43	"stage iii": ab,ti	31,468
	#44	stage iv: ab,ti	41
	#45	"stage iv": ab,ti	20,205
	#46	"stage iii/iv": ab,ti	6
	#47	"stage iii/iv": ab,ti	4,552
	#48	"stage iiib/iv": ab,ti	2
	#49	"stage iii/iva": ab,ti	1
	#50	stage* iii: ab,ti	32
	#51	stage* iii*: ab,ti	49
	#52	stage* iv: ab,ti	41
	#53	stage* iv*: ab,ti	52
	#54	stage iii*: ab,ti	47
	#55	stage iv*: ab,ti	46
	#56	inoperable: ab,ti	12,989
	#57	in* operable: ab,ti	13,465
	#58	unresectable: ab,ti	15,735
	#59	non* resectable: ab,ti	1,208
	#60	late* stage: ab,ti	84
	#61	late: ab,ti AND stage: ab,ti	41,346
	#62	metast*: ab,ti OR advance*: ab,ti	900,959
	#63	relaps* OR recurr* OR unresect* OR non?resect* OR in?operable OR non?operable OR un?operable OR advanc* OR metasta* OR late NEAR/2 stage	1,895,338
	#64	#34 OR #35	61,621
	#65	#36 OR #37 OR #38 OR #39 OR #40 OR #41 OR #42 OR #43 OR #44 OR #45 OR #46 OR #47 OR #48 OR #49 OR #50 OR #51 OR #52 OR #53 OR #54 OR #55 OR #56 OR #57 OR #58 OR #59 OR #60 OR #61 OR #62 OR #63	1,943,775
	#66	#64 AND #65	33,907
	#67	#33 AND #66	2,689
Language and year	#68	[1995–2013]/py AND [english]/lim	12,998,680
Final	#69	#67 AND #68	2,901

**Table 3:** Ovid/MEDLINE search strategy

**Ovid/MEDLINE search**			
**Category**	**Search**	**Query**	**Hits**
Design terms	#1	Randomized Controlled Trials as Topic/	100,690
	#2	Randomized Controlled Trial/	382,290
	#3	Random Allocation/	80,788
	#4	Double Blind Method/	129,303
	#5	controlled clinical trial.pt.	88,866
	#6	randomized controlled trial.pt.	382,290
	#7	Clinical Trial/	499,767
	#8	clinical trial.pt.	499,767
	#9	Clinical Trials as Topic/	173,590
	#10	1 or 2 or 3 or 4 or 5 or 6	631,570
	#11	7 or 8 or 9	602,541
	#12	((singl* or doubl* or treb* or tripl*) and (blind* or mask*)).ab,ti.	145,496
	#13	Placebos/	33,372
	#14	((random* and (clinical or control*) and trial) or (placebo* and ("randomly allocated" or (allocated and random*)))).mp. [mp = title, abstract, original title, name of substance word, subject heading word, keyword heading word, protocol supplementary concept, rare disease supplementary concept, unique identifier]	457,488
	#15	12 or 13 or 14	513,100
	#16	11 and 15	264,991
	#17	10 or 16	645,804
Disease terms	#18	((lung* or pulmon*) and (adenocarcinom* or squamous or "large cell" or "non-small cell")).ab,ti.	59,093
	#19	Carcinoma, Non-Small-Cell Lung/	34,861
	#20	(metastatic or advanced or stage or "stage 3" or stage4 or "stage 4" or stageIII or "stage III" or StageIV or "Stage IV" or Stage?III or "Stage ?III" or Stage?IV or "Stage ?IV" or "StageIII/IV" or "Stage III/IV" or "StageIII?/IV?" or "Stage III?/IV?" or "StageIII/StageIV" or "Stage III/Stage IV" or "StageIII?/StageIV?" or "Stage III?/Stage IV?" or inoperable or in?operable or unresectable or non?resectable or "late?stage" or (metast* or advance*)).ab,ti.	1,177,250
	#21	18 or 19	66,533
	#22	20 and 21	34,403
	#23	17 and 22	3,600
Exclusion terms	#24	case report.ab,ti.	201,610
	#25	review.pt.	1,893,388
	#26	letter.pt.	817,960
	#27	Clinical Trial, Phase I.pt.	15,867
	#28	Historical Article/	298,058
	#29	systematic review.ab,ti.	44,587
	#30	clinical review.ab,ti.	3,354
	#31	24 or 25 or 26 or 27 or 28 or 29 or 30	3,184,956
	#32	23 not 31	2,702
Rx terms	#33	drug therapy/ or treatment outcome/ or ("treatment" and "response").ab,ti. or ("drug" and "efficacy").ab,ti. or outcome assessment/	1,014,086
	#34	32 and 33	1,611
Final	#35	limit 34 to yr = "1995 -Current"	1,464

The following is a list of the conference databases that will be searched:

• American Association for Cancer Research, AACR

• American College of Radiation Oncology

• American Society for Radiation Oncology, ASTRO

• American Society of Clinical Oncology, ASCO

• Asia Pacific Lung Cancer Conference, APLCC

• Asia Pacific Oncology Summit, APOS

• Asian Oncology Summit, AOS

• Atualizacoes em Oncologia

• Australian Lung Cancer Conference, ALCC

• Austrian Society of Haematology and Oncology, ASHO

• The Association for Cancer Surgery, BASO

• Biennial Congress of the European Association for Cancer Research, EACR

• British Thoracic Oncology Group Conference, BTOG

• Clinical Oncology Society of Australia, COSA

• Cancer Symposium of the Society of Surgical Oncology, CSSSO

• Chicago Supportive Oncology Conference, CSOC

• Clinical Interventional Oncology, CIO

• Congres National de la Societe Francaise de Radiotherapie Oncologique, SFRO

• Congress of the European Society for Medical Oncology, ESMO

• Congress of the European Society of Surgical Oncology, ESSO

• Congress of the International Society of Oncology and Biomarkers, ISOBM

• Educational Cancer Convention Lugano of the European School of Oncology, ECCLU

• European Lung Cancer Conference, ELCC

• European Multidisciplinary Cancer Congress

• European Multidisciplinary Conference in Thoracic Oncology, EMCTO

• Hematology Oncology Pharmacy Association Annual Meeting, HOPA

• International Conference and Exhibition on Cancer Science and Therapy, IMPAKT

• International Conference of the Society for Integrative Oncology

• International Lung Cancer Congress

• International Symposium on Targeted Anticancer Therapies, TAT

• Italian Society of Surgical Oncology Conference

• Medical Oncology Group of Australia, MOGA

• Oncology Platform and Poster Presentation, CSM 2009

• Scientific Association of Swiss Radiation Oncology, SASRO

• Scientific Meeting of the International Society for Biological Therapy of Cancer

• Scientific Meeting of the Society for Immunotherapy of Cancer, SITC

• Symposium of the International Society of Oncology Pharmacy Practitioners

• UK Radiation Oncology Conference

• World Conference on Interventional Oncology, WCIO

• World Congress on Cancer Science and Therapy

### Eligibility assessment

To be eligible, published studies must meet the criteria outlined in Table 4. Briefly, eligible articles must report at least one of the following outcomes (overall survival, progression-free survival, quality of life, or toxicity) for patients with squamous NSCLC. Eligible articles must report data from randomized controlled trials published since 1995. Abstracts of all potentially eligible citations will be reviewed and excluded if it can be definitively stated that no eligibility criterion is met. All other publications will be considered potentially eligible. Full-text articles of all potentially eligible citations will be obtained and reviewed to determine final eligibility. The eligibility of both the abstracts and full-text articles will be assessed independently by two reviewers using the criteria and screening matrix presented in Table 4. If the two reviewers do not agree on the eligibility of an article, a third reviewer will serve as the tie breaker. Systematic reviews and other review articles will be scanned to ensure no eligible randomized controlled trials (RCTs) are missed.

**Table 4:** Eligibility criteria and screening matrix

**Eligibility criteria**			
Patients	Male or female patients with histologically or cytologicallyconfirmed squamous NSCLC		
	Study participants must have not received chemotherapy treatment prior to first-line chemotherapy for NSCLC at the time of randomization in the study		
Interventions	The study assesses a chemotherapy treatment in each of the study arms		
	No limits are placed on the type of chemotherapy used		
Outcomes	One or more of the following outcomes must be quantitatively reported in the publication: overall survival, progression-free survival, toxicity, or quality of life		
	At least one of the required outcome variables must be reported separately for patients with advanced or metastatic (stage III/IV) NSCLC that is of squamous histology		
Study design	RCTs		
Time frame	1995 to present		
**Ineligibility criteria**			
Interventions	Not first-line treatment with first-line defined as patients with no prior exposure to chemotherapy		
	Radiation therapy in the absence of concurrent chemotherapy in any treatment group		
Study design	Review articles, news, editorials, commentaries		
Time frame	Publication date prior to 1995		
**Matrix for patients with "squamous histology"**			
**Squamous inclusion obvious in abstract?**	**Squamous results obvious in abstract?**	**Inclusion**	**Comments**
Yes	Yes	Yes	
No	Yes	Yes	
Yes	No	Yes/No	Need full text to determine the inclusion
No	No	Yes/No	Need full text to determine the inclusion
**Only non-squamous inclusion obvious in abstract?**	**Squamous results obvious in abstract?**	**Inclusion**	**Comments**
Yes	Yes	Not possible case	
No	Yes	Yes	
Yes	No	No	
No	No	Yes/No	Need full text to determine the inclusion
**Abstract mentions just NSCLC as inclusion?**	**Squamous results obvious in abstract?**	**Inclusion**	**Comments**
Yes	Yes	Yes	
No	Yes	Yes/No	This may be multisite cancer study, need full text to determine the inclusion
Yes	No	Yes/No	Need full text to determine the inclusion
No	No	Noise in the search	Need full text to determine the inclusion
Multiple cancers	If mentions lung cancer	Yes/No	Need full text to determine the inclusion
	If does not mention any specific tumor types	Yes/No	Need full text to determine the inclusion
	If only mentions breast cancer or other types and does not mention lung cancer	Noise in the search	
**Matrix for "not first-line treatment"**			
**Condition**	**Line of treatment to be considered**	**Inclusion**	**Comments**
Naïve NSCLC patients	1st	Yes	
First- or front-line treatment	1st	Yes	
Untreated NSCLC patients	1st	Yes	
Metastatic chemo-naïve NSCLC patients	1st	Yes	
Chemo-naïve NSCLC patients	1st	Yes	
Second-line treatment	2nd	No	
(Rx)-resistant NSCLC patients	2nd	No	
Recurrent or progressive disease	2nd	No	
(Rx)-responder/non-responder patients	2nd	No	
If no clear information on line of treatment	NA	Yes/No	Need full text to determine the inclusion

### Data extraction and verification

In a process similar to that used for assessing eligibility, two reviewers will independently extract the data elements listed in Table 5 from each eligible article. These data are extensive and it is not expected, nor is it required, that all studies will report all data fields included. However, attempts to collect as extensive of data as possible will be made to increase the potential range of sensitivity and descriptive analyses. In addition to the data extraction, two reviewers will also assess bias using the Cochrane Risk of Bias Tool and will measure study quality using the Physiotherapy Evidence Database (PEDro) scale (see the "Assessment of bias and study quality" section). Data from both reviewers will be compared. If any data element does not match, the reviewers will meet and attempt to resolve the discrepancies. In cases of non-resolution, a third reviewer will be consulted. All rules and decision criteria used in the data resolution process will be recorded for quality assurance and methodological consistency purposes. To further ensure the accuracy of the extracted data, a subset of 10% of all extracted articles will be verified by an individual not involved in the data extraction process. In cases of error detection, the full database will be reviewed to ensure accuracy.

**Table 5:** Variables for data extraction

**Arm-phase-period information by study arm and overall**		
Arm	Unique arm number. Unique number for the treatment arm is a grouping variable that is used to highlight which outcome is in the same group of subjects	Integer, in case of sub-analysis arm use A.a format. Placebo = 0, for sub-arm 0.1
Number of study arms		1, 2, 3
Open label versus blinded		
Phase of study		1, 2, 3, or unknown
Objectives		OS, PFS, RR, TPD, etc.
Patients randomized	Number of patients randomized to the arm	Number value
Arm description	Description of the treatment arm usually includes the drug name, dose, and frequency	e.g., methotrexate 10 mg QW (once a week)
Sub arm analysis	Indicates if the analysis is in a subset of study arm	Yes, NA. Use all in case of AE or dropout data is reported for the randomized trial population
Arm comment	Comment referring to the arm	Comment in relevance to the understating of arm or NA
Study phase	Description of the specific phase within the overall study from which the data is derived	Lead-in, active, follow-up
Study phase description	Qualifies the "Study Phase" field with any additional information deemed necessary or helpful for that arm	e.g., open-label follow-up
Phase duration	Length of time of the study phase from which the data is derived for the arm	Time
Phase duration unit	Time unit for phase duration for the arm	Units
Phase comment	Comment concerning the study phase	Any comment that is relevant to the understanding of the phase or NA
Period	Used if necessary to separate crossover periods within a crossover trial	If the phase has multiple periods, the number of the period. Integer in sequence, or NA
Period description	Used to qualify the "Period" field with any additional information deemed necessary or helpful	e.g., treatment A, titration, maintenance, NA
Period duration	Length of time of the period in a study phase from which the data is derived	Time, NA
Period duration unit	Time unit for period duration	Units, NA
Period comment	Comment concerning the period	Any comment that is relevant to the understanding of the period or NA
Repository	Description	Data entry standards
**Demographics and medical history information at baseline by study arm and overall—adjusted and unadjusted**		
Age	Mean (or median) age in years of patient population or treatment arm population	Age in years or NR if not mentioned specifically or clearly in the trial
Percent female	Percent of females in the patient population or treatment arm population	Percent or NR if not mentioned specifically or clearly in the trial
Weight	Mean body weight of the patient or treatment arm population	Weight in kg, normalize if needed or NR if not mentioned specifically or clearly in the trial
Height	Mean height of the patient or treatment arm population	Height in cm, normalize if needed or NR if not reported
BMI	Mean body mass index of the treatment arm population	BMI in kg/m^2^, normalize if needed or NR if not reported in the trial
DBP	Mean (or median) diastolic blood pressure	mmHg
SBP	Mean (or median) systolic blood pressure	mmHg
Inclusion	Description of treatment arm or sub-arm inclusion criteria under the trial protocol	e.g., for sub-group females only, or NR if not mentioned specifically or clearly in the trial
Exclusion	Description of treatment arm or sub-arm exclusion criteria under the trial protocol	e.g., for sub-group exclusion of females with child-bearing potential, or NR if not mentioned specifically or clearly in the trial
Ethnic white	Percent of the ethnic population who are whites or Caucasian in the trial	Percent or NR if not mentioned specifically or clearly in the trial
Ethnic black	Percent of the ethnic population who are black in the trial	Percent or NR if not mentioned specifically or clearly in the trial
Ethnic Hispanic	Percent of the ethnic population who are Hispanic in the trial	Percent or NR if not mentioned specifically or clearly in the trial
Ethnic Asian	Percent of the ethnic population who are Asian in the trial	Percent or NR if not mentioned specifically or clearly in the trial
Ethnic other	Percent of the ethnic population who are other in the trial	Percent or NR if not mentioned specifically or clearly in the trial
Primary disease	Primary disease being studied	
Percent current smokers	Percent of the population who are current smokers	Percent or NR if not mentioned specifically or clearly in the trial
Percent previous smokers	Percent of the population who are previous smokers	Percent or NR if not mentioned specifically or clearly in the trial
Percent adenocarcinoma type	Percent subjects with NSCLC adenocarcinoma type	Percent or NR if not mentioned specifically or clearly in the trial
Percent squamous cell carcinoma type	Percent subjects with NSCLC squamous cell carcinoma type	Percent or NR if not mentioned specifically or clearly in the trial
Percent non-squamous		
Percent NSCLC stage 0**/**I**/**II	Percent subjects with NSCLC stage 0 or I or II	Percent or NR if not mentioned specifically or clearly in the trial
Percent NSCLC stage III	Percent subjects with NSCLC stage III	Percent or NR if not mentioned specifically or clearly in the trial
Percent NSCLC stage IV	Percent subjects with NSCLC stage IV	Percent or NR if not mentioned specifically or clearly in the trial
Percent NSCLC stage III/IV total	Total percent of subjects with NSCLC stages III or IV	Percent or NR if not mentioned specifically or clearly in the trial
Percent ECOG status 0	Percent subjects with Eastern Cooperative Oncology Group performance status scale 0	Percent or NR if not mentioned specifically or clearly in the trial
Percent ECOG status 1	Percent subjects with Eastern Cooperative Oncology Group performance status scale 1	Percent or NR if not mentioned specifically or clearly in the trial
Percent ECOG status 0/1 total	Percent subjects with Eastern Cooperative Oncology Group performance status scales 0 or 1	Percent or NR if not mentioned specifically or clearly in the trial
Percent Karnofsky status ≥80	Percent subjects with Karnofsky’s index of performance status >80%	Percent or NR if not mentioned specifically or clearly in the trial
Percent WHO performance status 0/1	Percent subjects with WHO performance status scale 0 or 1	Percent or NR if not mentioned specifically or clearly in the trial
Num of metastatic lymph nodes	Mean or median number of metastatic lymph nodes	NR if not mentioned specifically or clearly in the trial
Percent metastatic L-node positive	Percent subjects who are lymph node positive or with metastatic lymph nodes	Percent or NR if not mentioned specifically or clearly in the trial
Percent bone metastasis	Percent subjects with bone metastasis	Percent or NR if not mentioned specifically or clearly in the trial
Percent brain metastasis	Percent subjects with brain metastasis	Percent or NR if not mentioned specifically or clearly in the trial
Percent liver metastasis	Percent subjects with lung metastasis	Percent or NR if not mentioned specifically or clearly in the trial
Percent other metastasis	Percent subjects with other metastatic organs	Percent or NR if not mentioned specifically or clearly in the trial
Percent metastatic organ sites 1	Percent subjects with one metastatic organ or site involved	Percent or NR if not mentioned specifically or clearly in the trial
Percent metastatic organ sites 2	Percent subjects with two metastatic organs or sites involved	Percent or NR if not mentioned specifically or clearly in the trial
Percent metastatic organ sites >3	Percent subjects with three or more metastatic organs or sites involved	Percent or NR if not mentioned specifically or clearly in the trial
Percent hemoglobin <11.5 g/dl	Percent subjects with baseline hemoglobin levels <11.5 g/dl	Percent or NR if not mentioned specifically or clearly in the trial
Patient demographic comments	Any pertinent demographic comments that are not dealt by other variables	Any comment that may be relevant to the understanding of the demographic characteristics of the patient population
Percent previous surgery	Subjects with previous treatment for NSCLC as complete or partial surgery. Procedures include wedge resection (removal of part of a lobe), segmentectomy (removal of an anatomic division of a particular lobe of the lung), lobectomy (one lobe), bilobectomy (two lobes), or pneumonectomy (whole lung)	Percent or NR if not mentioned specifically or clearly in the trial
Percent previous radiotherapy	Percent subjects with previous radiotherapy as treatment for NSCLC	Percent or NR if not mentioned specifically or clearly in the trial
Percent previous chemotherapy	Percent subjects with previous chemotherapy as treatment for NSCLC	Percent or NR if not mentioned specifically or clearly in the trial
Comorbidities		At baseline and by treatment arm
Percent comorbidities		At baseline and by treatment arm;% or NR
Percent previous platinum		
Percent no previous treatment	Percent subjects with no treatment for NSCLC	Percent or NR if not mentioned specifically or clearly in the trial
Previous treatment comments	Comments regarding the previous treatment	Any comment that may be relevant to the understanding of the previous NSCLC treatment in this record, NA if no comments
**Pharmacological therapy information**		
**Repository**	**Description**	**Data entry standards**
Primary NSCLC therapy	Name of primary drug therapy used in this arm at that time point	NSCLC drug, e.g., cisplatin, docetaxel
Primary NSCLC dose	Randomized daily dose at time of outcome. Please note that this is the dose the patients were receiving when the observation is made (not the first randomized dose). If the treatment is switched at the time of observation, record the prior treatment the patients were getting just before the observation was made	Total daily dose at the time of observation
Primary NSCLC dose achieved	Average daily dose during assessment period or for the total treatment period	Average daily dose achieved. Specifically useful for dose titration and crossover trials, NA for the fixed dose trials as both dose achieved and total daily dose do not vary
Primary NSCLC dose unit	Unit of total daily or average dose achieved	Unit, NR if not reported
Primary NSCLC dose freq/cycle		
Primary Rx days of administration		e.g., d1, d8
Primary therapy duration and route of administration		e.g., 10 min i.v. infusion
Primary NSCLC Rx cycle duration		
Primary NSCLC Rx number of cycles		
Primary NSCLC formulation	Special treatment formulation	Only specialized formulations like IR, CR, SR
Primary NSCLC therapy status	Indicates whether the observation refers to the first, continuing or last dose of the therapy	Start = first dose starts on at this time, continuing = treatment is continuing at this time, end = treatment has been discontinued at this time (last dose)
Primary NSCLC dose comments	Comment regarding the dosing of primary NSCLC treatment	Any comment that may be relevant to the understanding the dosing of the primary treatment in this record, NA if no comments
Combo NSCLC therapy	Name of secondary NSCLC therapy used in this arm in addition to the primary treatment at that time point	NSCLC drug, e.g., PTH NA if no secondary NSCLC therapy
Combo NSCLC dose	Randomized daily dose of the secondary NSCLC therapy at time of outcome. Refer to the dose description of primary NSCLC dose	Total daily dose at the time of observation, NR if not reported and NA if no secondary NSCLC therapy
Combination NSCLC dose achieved	Average daily dose of the secondary NSCLC therapy during assessment period or for the total treatment period	Average daily dose achieved. Specifically useful for dose titration and crossover trials, NA for the fixed dose trials as both dose achieved and total daily dose do not vary
Combination NSCLC dose unit	Unit of total daily or average dose achieved for the secondary NSCLC therapy	Unit, NR if not reported or NA if no secondary NSCLC therapy
Combination NSCLC dose reg	Frequency of secondary NSCLC therapy being administered	QD, BID, etc., NA if no secondary NSCLC therapy
Combination NSCLC dose freq/cycle		
Combination Rx days of administration		e.g., d1, d8
Combination Rx duration and route of administration		e.g., 10 min i.v. infusion
Combination NSCLC Rx cycle duration		
Combination NSCLC Rx number of cycles		
Combination NSCLC dose comment	Comment regarding the dosing of secondary NSCLC therapy	Any comment that may be relevant to the understanding the dosing of the secondary treatment in this record, NA if no comments or no secondary NSCLC therapy
Concomitant medications		Baseline or by treatment arm
**Radiation therapy information**		
**Repository**	**Description**	**Data entry standards**
Radiation therapy type		
Radiation therapy comments		
**Assessment characterization**		
**Repository**	**Description**	**Data entry standards**
Assessment	Common name for assessment that this record refers to, e.g., PANSS	As in the assessments and conventions sheet
Assessment short form	Code for the assessment	As in the assessments and conventions sheet
Assessment comment	Any comment that describes the nature of the assessment	e.g., plasma glucose level, NA if no comments
Assessment location	Location from where the assessment value is taken or extracted from the manuscript	Table number, figure number, page number
Assessment category	Describes what the assessment value represented is, whether it is absolute, change from baseline (CFB), percent change from baseline (PCFB), or fraction of randomized patients with the event, count in case of tender or swollen joint counts	Absolute, CFB, PCFB, Frac, or Count
Assessment Stat parameter	The summary parameter of the assessment value	Mean, median, percent, NR if not reported
Stat population	Statistical population for which the efficacy/safety analyses were done and value reported	ITT, OC, completers, randomized, PPP (per protocol population: define), NR if not reported
Missing data treatment	Method used for handling with missing observations in computing the summary parameter	LOCF (last observation carried forward), none, NR if not reported and NA in case of completers
Scale lower limit	Scale lower limit	The lower limit of the scale for the assessment, NA if not applicable
Scale upper limit	Scale upper limit	The upper limit of the scale for the assessment, NA if not applicable
Assessment categories or words	Scale category description	Category that is associated with each point of the scale
Total levels	Total categories/points in the scale	The total number of categories associated with each point of the scale, e.g., 0 to 4 point scale
Total symptoms	Total symptoms in the scale	The total number of symptoms associated with the respective assessment, NA if not applicable
Total score lower limit	Lower limit of the scale, this is calculated as the number of levels multiplied with the lowest possible scale	Integer value, NA if not applicable
Total score upper limit	Upper limit of the scale, this is calculated as the number of levels multiplied with the highest possible scale	Integer value, NA if not applicable
Assessment level	For ordered categorical data "scales." Indicates which level in the categorical scale the assessment is referring to	Integer level from 1 to number of levels, if fractional responder type, enter responder threshold value, eg., ≥5% weight loss from baseline for total body weight assessment, etc., NA if not applicable
PROs	Scale, mean value, SD by group, time point	
**Time, assessment, and baseline value information**		
**Repository**	**Description**	**Data entry standards**
Assessment visit	Clinical visit at which the assessment is done	Visit 1 (usually baseline) is the first visit in the active phase. Lead in visits start at -1 and count backwards, NR if not reported
Assessment time reported	Time at which the assessment is done during the study and as reported in the manuscript	Visit 1 = baseline = time 0 and the lead in assessment time starts at -1 and count backwards
Assessment time unit reported	Unit for reported assessment time	Time unit as reported
Assessment time range reported	In case if the assessment values are average over a time interval	e.g., weeks 2 through 28 enter 2–28
Assessment time normalized	Normalized time in days at which the assessment is done during the study and as reported in the manuscript	The normalized time value using the normalized unit as days, e.g., 4 weeks = 28 days
Assessment time unit normalized	Unit for standard assessment time	Days is the standard unit
Assessment value	Assessment value reported at that time point	Assessment value as reported
Assessment unit	Assessment unit as reported	Assessment unit as reported, NA if not applicable
Assessment SE	SE of reported assessment value	SE as reported, NR if not reported
Assessment SD	SD of reported assessment value	SD as reported, NR if not reported
Assessment CI type		
Assessment lower CI		
Assessment upper CI		
Assessment value normalized	Assessment value converted into normalized assessment units	Still insert value here, report if normalized units are the same as the reported units
Assessment value unit norm	Normalized assessment value units	See assessments and conventions sheet for normalized assessment standard
Assessment SE normalized	Standard error of normalized assessment value	SE in the same units as normalized assessment, may need to be calculated from SD and *N*; if not provided, NA
Assessment SD normalized	Standard deviation of normalized assessment value	SD in the same units as normalized assessment, may need to be calculated from SE and *N*; if not provided, NA
Assessment CI type normalized		
Assessment lower CI normalized		
Assessment upper CI normalized		
Assessment number	Number of patients assessed at that time point and the value derived	Integer value, but for responders and dropouts, this value is calculated from the percentages reported in the trial
Assessment value comment	Comment pertaining to the assessment value that cannot be dealt by other variables	e.g., the assessment value is the mean of last 7 days of before each clinical visit, etc.
Hazard ratio	95% confidence intervals, progression-free survival, and overall survival—adjusted and unadjusted	
Baseline visit	Clinical visit at which the baseline assessment is done	Visit 1 = baseline = time 0, NR if not reported
Baseline time	Time at which the baseline assessment is done	Visit 1 = baseline = time 0
Baseline time unit	Unit for reported baseline time	Time unit as reported
Baseline time normalized	Normalized time in days at which the baseline is done during the study and as reported in the manuscript	The normalized time value using the normalized unit as days, e.g., -4 weeks = -28 days
Baseline time unit normalized	Unit for standard baseline time	Days is the standard unit
Baseline value	Absolute baseline value for that assessment	Absolute baseline value
Baseline value unit	Assessment unit as reported	Assessment unit as reported, NA if not applicable
Baseline SE	SE of the absolute baseline value	SE as reported, NR if not reported
Baseline SD	SD of the absolute baseline value	SD as reported, NR if not reported
Baseline CI type		
Baseline lower CI		
Baseline upper CI		
Baseline value normalized	Baseline value converted into normalized baseline units	Still insert value here report if normalized units are the same as the reported units
Baseline value unit normalized	Normalized baseline value units	See assessments and conventions sheet for normalized assessment standard
Baseline SE normalized	Standard error of normalized baseline value	SE in the same units as normalized baseline, may need to be calculated from SD and *N*; if not provided, NA
Baseline SD normalized	Standard deviation of normalized baseline value	SD in the same units as normalized baseline, may need to be calculated from SE and *N*; if not provided, NA
Baseline CI type normalized		
Baseline lower CI normalized		
Baseline upper CI normalized		
Baseline *N*	Number of patients from which the baseline value is derived	Integer
Baseline value comment	Comment pertaining to the baseline value that cannot be dealt by other variables	e.g., the baseline value is the mean of last 7 days of the run in period
**Reference specifications**		
**Repository**	**Description**	**Data entry standards**
Ref code	Numerical code assigned for the literature citation. Maps the record to the assessment details	Integer
Protocol or trial number	Protocol ID or the number of the trial report	As reported, NA if not applicable
Date modified	Date of initial entry or subsequent modification of the data point	mmddyy format
Modified by	Initials of curator	
Modification comment	Any comment that is relevant to modification by the curator	Initial entry if new record, brief statement of change(s)
Copyright status	Provided by the client or procured by the service provider	Client provided or yes in case the manuscript is procured by the service provider
Author	Authors of publication	As reported
Journal	Journal name	Standard abbreviated forms can be used, generally as in the PubMed
Publication year	Year of the publication	Integer
Title	Title of the study	
Volume	Volume number of the publication	e.g., 180
Pages	Page numbers of the publication	e.g., 1–24
Trial name alias	Trial name that trial is commonly referred to	NR if not reported
Inclusion description	Provide description of inclusion criteria	Can be cut and paste from PDF, can be placed in an attached note
Exclusion description	Provide description of exclusion criteria	Can be cut and paste from PDF, can be placed in an attached note
Study design	Brief description of the study design	Parallel-fixed arm, dose escalation, effect titration, crossover, etc.
Location of the trial	Geographical location where the study is conducted	Primary nationalities list
Number of countries	Number of countries the study is conducted	Integer
Number of centers	Number of centers the study is conducted	Integer
Trial start date	Date when the trial started	mmddyy format
Trial end date	Date when the trial completed	mmddyy format
Placebo-controlled or active comparator	Was there a control group and was it placebo	PBO control/active comparator
Active comparator therapy	If this was an active comparator trial what was the comparator therapy	e.g., PTH
Percent randomized to placebo	Percent of subjects in the trial who are randomized to placebo	Integer
Add-on/washout study	Was the study drug added on to standardized background Rx, was background Rx washed out prior to starting primary Rx, or was standardized background therapy withdrawn once primary RX started	Add-On, Washout, Replacement, None
Study blind	Was the trial blinded for the treatment phase	Yes, double blind
Number of arms	Number of treatment arms the patients are randomized to	Integer
Arm description	Codes and description for arms	0 = placebo and others in sequence
Dose descriptions	Brief descriptions of the treatment drugs and the respective doses along with regimens received	0 = placebo, 1 = metformin 10 mg QW…
Dose ranging within study	Does the trial contain at least two primary treatment arms where different dose strengths were administered	Yes, No. Placebo does not count as a dose strength
Primary longitudinal data	Were multiple time values reported for the primary assessment endpoint	Yes, No.
Active phase trial duration	What was the duration of the active phase of the trial	Time, units, i.e., 3 weeks
Steady state effect achieved?	Does it appear that effect stabilized over time for primary endpoints	Yes, No. Not clear
Was there a lead-in phase?	Was there a standardized lead-in phase in the study other than a simple screening visit	Yes, No
Lead-in phase duration	If so, what was the duration	If yes, time, units, i.e., 6 months. If no, 0
Was there a follow-up phase?	Was there a standardized follow-up phase that at least some patients were enrolled in after the active phase ended	Yes, No
Duration of follow-up phase	If so, what was the duration	If yes, time, units, i.e., 6 months. If no, 0
Primary endpoint	What is the primary outcome or assessment reported in the trial	e.g., HBA1C
Secondary endpoints		
Other efficacy endpoints available	List of the other secondary efficacy or biomarker outcomes reported in the trial	e.g., HOMA
Most frequent AEs (incidence)	List the most frequently reported AE’s	e.g., vomiting, nausea, headache, dizziness
Adverse events	Grade,%, *n* by group, treatments for AEs, hospitalizations secondary to AEs and overall, ICU admissions secondary to AEs and overall	
Median and mean if reported	Progression-free survival and overall survival in months (TTPD, TTTF)	
Survival rates	Percent alive at *X* months	
Response rates	At what time?	
Toxicity	Number of individuals experiencing toxicity/treatment group	

### Analysis plan

A PRISMA (Preferred Reporting Items for Systematic Reviews and Meta-Analyses) diagram will be developed based on the search strategy and eligibility assessment to show the flow of included and excluded studies. The descriptive statistics from each trial of patients with squamous cell carcinoma will be included and described. These variables will include treatment group, number of patients, mean age (standard deviation), number and percent male, number and percent with stage IV disease, overall survival, progression-free survival, toxicity, and quality of life.

A network diagram visually describing existing treatments for squamous NSCLC will be created after all eligible studies have been identified. However, some publications may not present data in a format that allows them to be included in the study despite otherwise meeting eligibility criteria (e.g., mixed populations not reported separately, mixed histologies not reported separately, mixed lines of therapy not reported separately). In the case of a disconnected network resulting from the absence of data for the appropriate patient population, authors of such articles will be contacted and asked to provide the needed data from their publications that would enable connection to the studied network.

The primary purpose of this study is to perform indirect and direct comparisons of GC + N versus all identified comparators for overall survival (OS) and progression-free survival (PFS). Individual hazard ratios (HR) or median time-to-event (median time) and 95% confidence intervals (90% or 99% confidence intervals will be converted to 95%) for overall survival will be included in the network meta-analysis using a Bayesian approach that ensures the preservation of randomization in the network [10]. The HR will be used as the primary unit of analyses to evaluate differences in effect size between treatment groups. Data for analysis will be extracted directly from the text of each eligible article, calculated from data included in the text, or extrapolated from the Kaplan-Meier plot according to the method of Parmar and colleagues [11]. Graphs and figures will be digitized using TechDig software and/or *xyscan* tool (Debian, Inc) if necessary, and digitized values will be extracted.

Heterogeneity will be explored by comparing the fixed and random effects models to ensure that the network has good properties. Additionally, heterogeneity will be explored by visual inspection of forest plots. The consistency assumption will be tested by examining network diagrams to identify any closed "loops" where inconsistencies can occur. When the network is complex with multiarm trials, the "node-splitting" approach defined by Dias and colleagues [12] will be used to identify inconsistencies. Density plots of the posterior samples from models based on direct, indirect, and mixed evidence will be compared. In addition, the heterogeneity parameters (variance and standard deviation) and goodness of model fit measures (residual deviance and deviance information criterion (DIC), a Bayesian criterion for model comparison) between the direct and indirect models will be compared.

OS and PFS data will be analyzed using a log transformation of the HR and treating this as a continuous outcome. For studies with median time information, we will also use log transformation of the median time and treat this as a continuous outcome in sensitivity analyses. HRs are preferred summary statistics to median time per Michiels and colleagues [13], and hence, the analysis will utilize HR data for the primary outcome measure.

Ideally, the literature will provide values for log (HR) and the standard error (SE) for log (HR). If the SE for log (HR) is not available, an attempt will be made to estimate the missing value from the SE for median time, assuming an exponential distribution of survival time and log (HR) = -log (median time ratio). Alternatively, an estimate of the SE for log (HR) will be made on the basis of the number of subjects with events as specified below:

1. "MedianTime" will be converted into log (median time);

2. The SE for log (median time) is estimated as (log(upper confidence limit) - log (lower confidence limit))/2/quantile (confidence level) if a treatment arm has non-missing value for all three variables;

3. If confidence limit is missing, then the number of subjects with events can be used to estimate the standard error for log (median time) as 1/sqrt(*n*) for a treatment arm.

Individual odds ratios and/or toxicity rates for each grade 3–4 toxicity from each study will be included, respectively, in an NMA using a Bayesian approach that ensures the preservation of randomization in the network. Odds ratios will be calculated for studies reporting toxicity rates. Prior to creating the odds ratios, we will ensure that similar versions of toxicity scaling criteria have been used. Data for analysis will be directly extracted from the text of the article or calculated from data in the text.

A network meta-analysis of GC + N to all identified comparators will be conducted for health-related quality of life (HRQoL) measures (including EQ-5D and the Lung Cancer Symptom Scale (LCSS)) during and following therapy. The most common quality of life instruments as reported across studies will be analyzed. Initial analyses will be limited to those quality of life outcomes for which GC + N data are available. For each identified measure, a standardized mean difference in quality of life outcomes from each study will be included. First, the number of trials per HRQoL instrument will be determined. If the number of trials per HRQoL instrument is 2 or more, then these data will be analyzed. For each instrument, data will be assessed according to the guidelines for that particular instrument and then pooled across studies to determine the standardized mean difference.

A meta-regression will be conducted using the key covariates of patient age and stage of disease (percent of patients with stage IV), as these variables have prognostic value in squamous NSCLC. Additional covariates may be identified following the literature review and will be considered for inclusion in *post hoc* analyses to control for potential bias.

### Sensitivity analyses

We anticipate that some studies will not report all relevant data. In order that such studies can still be included in the analysis, we may consider imputing missing data using established methods as appropriate [14]. If imputation is made, the Bayesian model as described above will be used as the primary analysis and will be compared with analyses including the imputed values. Sensitivity analyses may be conducted to examine the effect of this method using an approach proposed by Carpenter and colleagues [15], which entails imputing missing data under a missing at random assumption, and then reweighting the imputed data to allow for non-random selection. Sensitivity analyses as outlined for OS and PFS will also be conducted for HRQoL; however, the use of disparate HRQoL instruments or assessment time points may result in an inability to evaluate the study endpoint. Sensitivity analyses will be performed to assess the robustness of the findings. At a minimum, the following analyses will be conducted if there are at least three studies available for analysis:

1. Repeat the meta-analysis using a frequentist approach;

2. HR only (primary aim) versus HR or median time;

3. By geographical site of study enrollment;

a. e.g., Western versus Eastern hemispheres

b. e.g., Americas versus Europe versus Asia

4. Limit to patients with stage IV disease;

5. Direct comparisons only;

6. By excluding phase II trials;

7. By age—studies with a mean age over the age of 70;

8. Limiting the analysis to high-quality studies (≥6) as determined by the PEDro scale;

9. Removing studies considered to be biased according to the Cochrane Risk of Bias Tool.

### Assessment of bias and study quality

The risk of bias will be appraised using the Cochrane Risk of Bias Tool ( http://www.cochrane-handbook.org). This tool was developed specifically to assess the internal validity of RCTs. It consists of the following seven criteria: 1) randomization generation, 2) allocation concealment, 3) blinding of outcome assessors, 4) blinding patients and personnel, 5) incomplete outcome data (i.e., withdrawals), 6) selective outcome reporting, and 7) other risks of bias. The final item will include fraudulent results, other methodological flaws in the RCTs, and the potential for bias.

To assess publication bias, the fail-safe *N* will be calculated. If the number of unpublished trials that may invalidate the findings is less than five, it will be noted in the conclusions as a potential limitation of the findings. If the number of unpublished trials to invalidate the findings is five or greater, it will be noted in the results. Furthermore, funnel plot analyses will also be conducted to provide a visual representation demonstrating where unpublished data may exist. This is planned to help guide the interpretation of the study findings and the direction of bias.

Quality of selected trials for inclusion in the review will be assessed. The PEDro quality scale, an 11-item scale designed for rating the methodological quality of randomized controlled trials [16], will be used to evaluate the quality of selected trials. Here the two reviewers will independently assess studies for methodological validity prior to inclusion. Identified studies that meet the inclusion criteria will then be grouped according to the class of statin used in the trial. High quality scores will be defined as a PEDro score ≥6 and low quality scores will be defined as a PEDro score <6.

Missing data are expected in the majority of data fields collected in this meta-analysis. In cases of missing data, heterogeneity will be tested on all outcome variables to ensure that studies are comparable. Forest plots will be created for OS, PFS, toxicity, and quality of life endpoints. In the case of non-overlapping confidence intervals, the research team will discuss the need for *post hoc* subgroup analyses.

## Discussion

The study design for this systematic review and meta-analysis is presented here to follow PRISMA standards. Industry-sponsored or industry-led studies are increasingly under scrutiny regarding transparency and risk of bias [17]. This study protocol has been designed prior to any knowledge of the study data or outcomes from existing published literature and is being disseminated in an attempt to provide the scientific community with the ability to evaluate the methods and plans of our study before it is conducted. The study protocol has been designed to meet PRISMA standards [18,19] and is being disclosed so that our methods can be retrieved and evaluated against the final analyses and interpretation of findings.

While it is almost impossible to fully anticipate the limitations of the data once they are obtained, this study has been designed in an attempt to pre-specify all primary analyses and sensitivity analyses to demonstrate the stability in results that may be discovered. However, it is possible that there will not be sufficient data to achieve all the pre-specified study aims or to complete all planned analyses. There are also possible limitations in the network connections. Unlike patients diagnosed with lung cancers of non-squamous histology, those with squamous NSCLC have not benefited from the same depth and breadth of research conducted to identify optimal treatment strategies. Therefore, via our search criteria, we are casting a wide net in the hopes of finding studies that not only investigate, but also report, outcomes for this histological subgroup.

Our ultimate goal is to provide reliable and trustworthy data regarding the comparative efficacy of necitumumab against other possible options for care so that decision makers can come to their own conclusions regarding the value of this molecule currently in development.

## Competing interests

All study authors disclose that they are employees of Eli Lilly and Company.

## Authors’ contributions

AD participated in the study design, development of the study protocol, drafting of the analysis plan, and writing of the manuscript and will be responsible for data and eligibility review. JB conceived of the study design, review of the study protocol, and substantive input to the manuscript. FN developed the study protocol analysis plan and will be responsible for final analyses. LZ developed the study protocol analysis plan and will be responsible for final analyses. ZC developed the study protocol analysis plan and will be responsible for final analyses. SA will be responsible for data and eligibility review. LB and JT conceived of the study design and development of the study protocol. LH conceived of the study design, development of the study protocol, writing of the manuscript, and drafting of the analysis plan and will be responsible for data and eligibility review. All authors reviewed and approved the final version of the manuscript.
